# Untangling the Cooperative Role of Nuclear Receptors in Cardiovascular Physiology and Disease

**DOI:** 10.3390/ijms22157775

**Published:** 2021-07-21

**Authors:** Ana Paredes, Rocio Santos-Clemente, Mercedes Ricote

**Affiliations:** Myocardial Pathophysiology Area, Centro Nacional de Investigaciones Cardiovasculares (CNIC), 28029 Madrid, Spain; aparedes@cnic.es (A.P.); rocio.santos@externo.cnic.es (R.S.-C.)

**Keywords:** heart, cardiomyocyte, transcription, nuclear receptors, RXR, PPAR, cardiovascular disease, myocardial infarction, atherosclerosis

## Abstract

The heart is the first organ to acquire its physiological function during development, enabling it to supply the organism with oxygen and nutrients. Given this early commitment, cardiomyocytes were traditionally considered transcriptionally stable cells fully committed to contractile function. However, growing evidence suggests that the maintenance of cardiac function in health and disease depends on transcriptional and epigenetic regulation. Several studies have revealed that the complex transcriptional alterations underlying cardiovascular disease (CVD) manifestations such as myocardial infarction and hypertrophy is mediated by cardiac retinoid X receptors (RXR) and their partners. RXRs are members of the nuclear receptor (NR) superfamily of ligand-activated transcription factors and drive essential biological processes such as ion handling, mitochondrial biogenesis, and glucose and lipid metabolism. RXRs are thus attractive molecular targets for the development of effective pharmacological strategies for CVD treatment and prevention. In this review, we summarize current knowledge of RXR partnership biology in cardiac homeostasis and disease, providing an up-to-date view of the molecular mechanisms and cellular pathways that sustain cardiomyocyte physiology.

## 1. Introduction

Cardiovascular disease (CVD) is a heterogeneous group of heart and blood-vessel–related pathologies that includes coronary heart disease, cerebrovascular disease, peripheral arterial disease, inherited or acquired cardiomyopathy, and thrombosis. Acquired CVD is currently a global emergency due to its increasing incidence and the high prevalence of CVD risk factors in the general population. According to World Health Organization figures, CVD kills 17.9 million people every year, making it the leading cause of death in the world, responsible for approximately 31% of all deaths worldwide [1]. Over the past two decades, global CVD mortality has declined by almost 21%; however, this decline has been concentrated exclusively in developed economies, whereas CVD mortality in low-income and middle-income countries has increased [2]. CVD usually manifests in the context of a combination of several distinct risk factors. The prevalence of these risk factors varies between different age groups, but the most frequent risk factors at all ages are smoking, physical inactivity, unhealthy diet and obesity, hypertension, diabetes, hyperlipidemia, and alcohol or drug abuse [3]. CVD onset is also linked to environmental factors that are independent of personal behavior have also a big relevance for the onset of CVD [4]. These risk factors can ultimately culminate in atherosclerosis, acute ischemic events such as myocardial infarction (MI) and stroke, heart failure (HF), arrhythmias, and other chronic cardiac conditions [5]. CVD, both congenital and acquired, constitutes a major global public health problem requiring the development of novel and more effective treatments and prevention strategies. Although CVD pathophysiology has been widely studied, there is still a need for better understanding of its underlying molecular mechanisms, with many studies suggesting an important role played by nuclear receptors (NR). Here, we summarize current knowledge about class II NR function in biological processes essential for cardiovascular (CV) biology in health and disease, with a specific focus on the critical integrative functions of retinoid X receptors and their partnerships.

## 2. Nuclear Receptors

Nuclear receptors (NR) are a superfamily of transcription factors activated by lipid-soluble ligands, allowing them to act as intracellular integrators of hormonal and other extracellular stimuli [6]. Some NRs, known as orphan receptors, have no clearly identified ligand and yet have well-established functions [7]. In the nucleus, NRs directly modulate essential transcriptional programs by either promoting or repressing targeted gene expression [6]. NRs thus have the ability to translate physiological signals into complex transcriptional networks that result in a range of distinct effects. NRs play key roles in homeostasis and disease and are considered master regulators of transcription in several biological processes, including cell proliferation, differentiation, metabolism, reproduction, and circadian rhythms [6,8]. In humans, 20 of the 48 identified NR members have been found to have pathogenic variants directly associated with disease [9]. The essential roles of NRs in regulating pathophysiological mechanisms and their ability to bind small molecules together make them promising targets for drug development. 

NRs contain four functional domains, known as the A-B domain (N-terminal domain), C domain (DNA-binding domain [DBD]), D domain (hinge domain), and E domain (ligand-binding domain [LBD]). These domains regulate NR activity through post-transcriptional modifications (PTM) and protein-protein interactions (PPI) with co-regulators. The N-terminal A-B domain, which is less conserved and less well characterized than the other domains, plays an important role in ligand-independent transactivation. The DBD and LBD both participate in dimerization, but the ability of NRs to form homodimers of heterodimers is critically determined by the DBD. The LBD contains binding pockets for ligands and co-regulator proteins. The DNA sequences for NR dimer binding are known as DNA response elements (DREs) and generally consist of imperfect palindromic sequences or half-sites forming direct or inverted repeats [10].

The mechanism of action of NRs generally involves a cis-repressive function upon binding to target DREs. Ligand binding triggers conformational changes in the NR that allow the release of co-repressors and the recruitment of co-activators, leading to active transcription of target genes [6]. Some NRs (such as peroxisome proliferator-activated receptors [PPAR] and liver X receptors [LXR]) can inhibit inflammatory responses by interacting with other classes of signal-dependent transcription factors to prevent target gene expression [11]. This mechanism, known as trans-repression, in independent of DRE binding, since the NR associates with the locus indirectly.

The NR superfamily are classified into four subgroups based on DNA-binding motif structure, ligand specificity, and dimerization features. Class I NRs (steroid receptors) function as homodimers. Class II NRs include the retinoid X receptors (RXRs) and all the NRs which necessarily form heterodimers with RXRs. Class III NRs (homodimeric orphan receptors) are thought to act as homodimers but independently of ligand binding. Class IV NRs (monomeric orphan receptors) also regulate transcription independently of ligand activation but as monomers [12].

## 3. Retinoid X Receptors

Retinoid X receptors are unique among NRs in being essential structural partners for every class II NR, forming obligate heterodimers. Historically, RXRs were considered silent receptors with no transcriptional activity. However, the finding that RXR-containing heterodimers can be activated by RXR-specific ligands confirmed that RXRs are important transcriptional regulators involved in many distinct pathways [13]. RXRs can also form active RXR-RXR homodimers, confirming their inherent potential to modulate specific transcriptional networks [13,14]. Depending on their mode of activation, RXR-containing heterodimers can be classified as permissive, non-permissive, or conditionally permissive [13]. Permissive heterodimers can be activated both by RXR-specific ligands and by partner-specific ligands, permitting additive or synergistic effects. Permissive RXR partners comprise PPARs, LXRs, pregnane X receptor (PXR), farnesoid X receptor (FXR), Nurr1, and Nur77. Non-permissive heterodimers are activated only by specific agonists of the partner NR, with the RXR performing structural functions. This group includes vitamin D receptor (VDR) and thyroid receptors (TRs) [15]. Some heterodimers, such as retinoid acid receptors (RAR), are classed as conditionally permissive because their full transcriptional activity requires the presence of the RXR ligand. 

RXR-containing heterodimers (RXR partnerships) display a vast cistrome, genome-wide binding locations, in the CV system. Thus, they are considered as some of the most important transcriptional regulators in cardiac physiology and CVD. NRs are crucial effectors in many cell types, including macrophages, hepatocytes, and adipocytes [13,16], and cardiac NRs are implicated in cardiogenesis [17], the heart conduction system [18], and energy homeostasis and mitochondrial biogenesis [19] (Table 1).

RXRs specifically respond to *9-cis* retinoic acid (9cRA) and endogenous fatty acids (FA) [15,91,92]. The identification of natural RXR ligands is still a long matter of study. In 2000, The Perlmann group reported docohexanoic acid (DHA), a long-chain polyunsaturated FA, as a brain-derived RXR-activating factor [20]. Additionally, John Welch’s laboratory has recently described long-chain FA C24:5 as a physiological RXR ligand in hematopoietic cells [21]. The fact that this molecule is increased under stress conditions highlights the complex dynamics to which RXR signaling is subjected. 

RXRs occur in three isoforms encoded by distinct genes: RXRα (NR2B1), RXRβ (NR2B2), and RXRγ (NR2B3) [93]. These isoforms show tissue specificity, and almost all cells in the body are believed to express at least one, underlining the importance of RXRs in orchestrating transcriptional programs. RXRα and RXRβ are the main isoforms expressed in the developing heart, whereas RXRγ expression is restricted to postnatal cardiomyocytes [93]. 

The first study to link RXRα function to cardiac development came from the laboratory of Ron Evans, who reported that systemic lack of RXRα results in embryonic death at developmental stages E13.5–E16.5 due to severe myocardial malformations including ventricular septal, atrioventricular cushion, and conotruncal ridge defects, resulting in systolic dysfunction [94,95]. In a separate study, RXRα-knockout (KO) cardiomyocytes showed sustained expression of the atrial-like phenotypic marker myosin light chain 2 [94]. Heterozygous RXRα-KO embryos showed an intermediate phenotype, consisting of a predisposition to trabecular and papillary muscle defects, atrioventricular cushion defects, and pulmonary stenosis with no effect on survival [96]. Further analysis revealed that only one copy of RXRα gene is required for proper embryogenesis and survival [97]. Since the developing heart expresses the RXRα and RXRβ isoforms, double RXRαβ-KO mice were also investigated. Systemic RXRαβ-KO embryos did not survive beyond E6.5 due to abnormal placentogenesis, preventing the study of the combined contribution of RXRα and RXRβ to cardiac development. The phenotype of systemic RXRβ-KO mice features defects in spermatogenesis regardless cardiac alterations [98], whereas systematic RXRγ-KO mice develop normally and show no physiological alterations [97].

The role of RXRα in the heart has been investigated using cardiac-specific RXRα-KO mice. Surprisingly, heart function was unaffected in myocardium-specific and endocardium-specific RXRα-KO mice, excluding a cell-autonomous role in these cardiac layers [99,100]. Myocardial defects were detected in epicardium-specific RXRα-KO mice; however, these did not fully recapitulate the defects seen in systemic RXRα-KO embryos [100]. This study established the existence an RXRα-driven paracrine mechanism through which the epicardium promotes myocardial growth and coronary vessel formation by activating the Wnt9b/B-catenin pathway. Epicardial Wnt9b and FGF2 were highlighted as RXRα responsive genes, which promoted epithelial-to-mesenchymal transition (EMT) in a cell-autonomous manner. Subsequently, myocardial B-catenin stabilization and FGF2 synthesis. induced cardiomyocyte proliferation [100]. The non-cell autonomous role of RXRα in embryonic cardiomyocytes is further supported by the finding that RXRα cardiac-specific overexpression on an RXRα-null background did not prevent myocardial hypoplasia or fetal lethality [101]. Targeted mutation of the RXRα LBD (AF2) did not reproduce the phenotype of RXRα-KO embryos phenotype, suggesting that RXRα functions during heart development as a silent transcriptional partner [102]. Interestingly, in silico analysis identified RXRα and RXRγ as candidate mediators of cardiomyocyte maturation throughout life [37].

The role of RXRα in adult cardiac homeostasis and disease has not been explored in depth; however, several studies have provided insights into the role of RXRs during cardiac stress. In vitro assays in rat H9c2 ventricular cells subjected to hypoxia/reoxygenation revealed that 9cRA pretreatment increased cell viability, reduced the apoptosis ratio, and stabilized mitochondrial membrane potential [24]. The 9cRA pretreatment prevented abnormal levels of Bcl-2, Bax, or cleaved caspase-9, well-known mediators of apoptosis signaling. H9c2 cardiomyocyte expression of RXR protein is downregulated by hydrogen peroxide (H_2_O_2_) [23], a pro-apoptotic signal linked to cardiac dysfunction [25]. In line with these results, treatment with 9cRA or the synthetic RXR agonist LGD1069 (bexarotene) attenuated oxidative-stress–induced cell damage and loss of mitochondrial membrane potential [23]. In the failing heart, oxidative-stress activation of angiotensin-II (AngII) provokes detrimental cardiac remodeling and function [103]. Bexarotene treatment of cultured rat aortic smooth muscle cells blocked AngII-dependent inflammation and p38 stress mitogen-activated protein kinase (MAPK) activation, suggesting a protective role of RXR activation in cardiac disease [104].

Growing evidence suggests that RXR transcriptional circuits play a key role in regulating cardiac metabolism. RXRα expression is reduced in a dog model of severe HF, and this is accompanied by substantial alterations in fatty acid oxidation (FAO) [26]. Moreover, high glucose levels inhibit ligand-induced RXRα promoter activity in neonatal cardiomyocytes and promote apoptosis via JNK and oxidative-stress signaling [105]. 

Cardiac function is usually disrupted in diabetes, manifesting as diabetic cardiomyopathy (DiCM). As the affected hearts are unable to consume glucose, lipid use is markedly increased, which ultimately unbalances energy homeostasis and leads to contractile dysfunction. In this setting, dysregulated oxidative stress, inflammatory signals, and the AngII pathway converge. In a mouse model of noninsulin-dependent diabetes and obesity, the RXR agonist LG100268 was shown to act as insulin sensitizer, maintaining normal glucose and triglycerides levels [106]. Moreover, RXR activation by bexarotene attenuated diabetes-induced cardiac dysfunction by improving glucose tolerance and insulin resistance in Zucker diabetic fatty rats (ZDF) [27]. This finding is supported by work by Chai et al., who recently demonstrated that bexarotene inhibits myocardial fibrosis by modulating the LKB1/p70S6K signaling pathway in diabetic rats [28]. The protective action of bexarotene through LKB1/p70S6K is also conserved in spontaneously hypertensive rats (SHR), where it significantly reduces left ventricular hypertrophy without affecting blood pressure [107]. The cardioprotective action of the rexinoids isotretinoin and bexarotene was highlighted in a recent study showing that activation of endothelial RXRα protects against anthracycline-induced cytotoxicity in zebrafish [108]. 

The beneficial role of bexarotene has also been explored in the context of atherosclerosis. Lalloyer et al. demonstrated that the atheroprotective action of bexarotene in a dyslipidemic mouse model operates through non-cardiac effects, including an increase in the intestinal absorption of cholesterol and enhanced macrophage lipid efflux [29]. The presence of side-effects in this model underlines the need to develop tissue-specific RXR-targeted treatments for atherosclerosis. Another synthetic RXRα modulator, K-80003, was recently shown to attenuate atherosclerotic plaque progression and destabilization, macrophage infiltration, and inflammation in ApoE-null mice [30]. The authors proposed a non-canonical molecular mechanism by which K-80003 targets RXRα-mediated autophagy and inflammation. 

**Figure 1:**
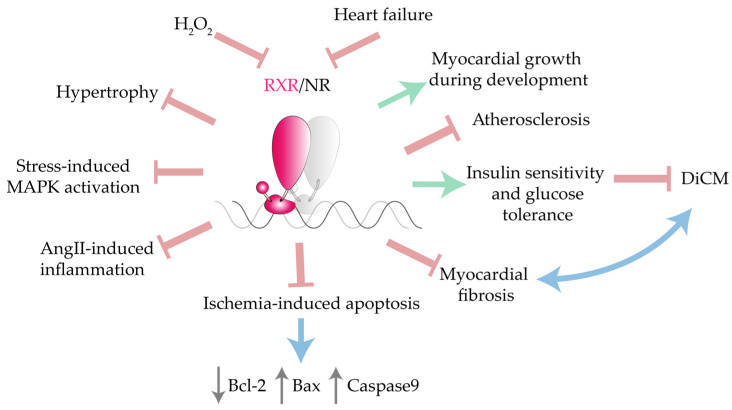
Cardiac-related RXRs function. Schematic representation of physiological and pathophysiological cardiac-related processes where RXR functioning is involved. Inhibitory signals for RXR expression are also depicted. Blue arrows denote homeostatic regulation. Green arrows denote induction. Red arrows denote inhibition. Grey arrows indicate physiological changes in protein levels. RXR, retinoid X receptor. NR, nuclear receptor. MAPK, mitogen-activated protein kinase. AngII, angiotensin II. DiCM, diabetic cardiomyopathy. Bcl-2, B-cell lymphoma 2 gene. Bax, Bcl-2-associated X protein gene.

These studies collectively suggest that RXRs are essential controllers of cardiac development, homeostasis, and disease (Figure 1). The involvement of RXRs in cardiomyocyte and non-cardiomyocyte physiology makes RXR-based pharmacological strategies a promising approach to CVD. 

## 4. Retinoic Acid Receptors

Retinoic acid receptors (RAR) are, together with RXRs, the main transducers of vitamin A in the body. Vitamin A is biologically inactive and is converted into several metabolic derivatives in the cell. The specific RAR ligand is all-*trans* retinoic acid (ATRA), which is produced from vitamin A by retinal dehydrogenase (RALDH) [109]. In the 1980s, three RAR isoforms were discovered: RARα (NR1B1) [110], RARβ (NR1B2) [111], and RARγ (NR1B3) [112]. These RAR isoforms have distinct tissue expression patterns, but almost every cell in vertebrate organisms is believed to have the ability to respond to retinoids, through either RARs or RXRs [113]. 

Retinoid signaling from dietary vitamin A is an essential driver of mammalian heart development. Initial studies showed that fetal vitamin A deficiency (VAD) causes defective cardiac development. In separate studies, Lohnes et al. and Mendelsohn et al. demonstrated that several combinations of systemic RAR double mutants show heart abnormalities, suggesting that these NR were the main retinol sensors. However, given that single RAR deletion did not reproduce fetal VAD syndrome, a redundant effect among RAR isoforms was proposed [114,115]. 

Mouse, zebrafish, and chicken models of defective vitamin A signaling have revealed that retinoic acid (RA) is required for several stages of heart development. At early stages, RA ensures proper looping of the heart tube by defining cardiogenic regions within the second heart field (SHF) and correct formation of the outflow tract. At later stages, RA signaling in the epicardium is important for myocardial proliferation and, therefore, for ventricle development and maturation. In addition, RA is crucial for the epicardial EMT required for coronary vessel formation [22]. 

A specific contribution of RAR to these processes has not been clearly defined, and studies of RXR mutants indicate that retinoid signaling through RXRs plays a major role in cardiogenesis [22,109]. Nevertheless, there is strong evidence for the action of RARs in cardiogenesis. Experiments in RALDH2-deficient mice have directly linked cardiac defects to ATRA availability, with mutant mice showing heart morphogenesis defects affecting heart tube looping and ventricular chamber development [32]. A more recent study showed that epicardial cell function is impaired by pharmacological inhibition of RALDH [31], suggesting that RAR activation by ATRA participates in the crucial RA signaling in the epicardium. Heart tube looping and other defects are also seen upon deletion of the ATRA-catabolizing enzyme CYP26 in mice [116,117]. Moreover, coronary vascular development and epicardial function are impaired in mice lacking retinaldehyde reductase (DHRS3), which prevents excessive RA signaling by reducing retinaldehyde to retinol [31,118]. These observations support evidence that excessive RA availability has teratogenic effects in diverse mouse models.

Despite the limited research into the role of RAR in the adult heart, there is evidence for a role of RA signaling in CVD [119]. A cardioprotective effect is suggested by ATRA supplementation experiments in animal disease models. Early evidence for this was an anti-apoptotic action of RA in cultured rat cardiomyocytes subjected to oxidative stress, operating through RARs and RXRs [33]. RAR activity is increased in a luciferase-reporter mouse model of MI and is linked to the induction of an RAR transcriptional program [34]. The same study also detected increased cardiac retinol levels and showed that ATRA specifically inhibited fibroblast proliferation in vitro, suggesting that RAR activation supports cardiac remodeling after MI. 

High-throughput screening in a human induced pluripotent stem-cell model has identified ATRA as a potent inducer of proliferation in cardiac progenitor cells [120], suggesting that RARs may play a role in heart regeneration. Interestingly, age-induced cardiac electrophysiology abnormalities in rats are prevented by treatment with a novel microalgae-derived carotenoid that acts by increasing RARα expression and shows evidence of RARα in silico [121]. Another recent study in human samples [36] showed that ATRA suppresses the calcification of coronary artery and aortic vascular smooth muscle cell (VSMC), an important driver of coronary heart disease and peripheral arterial disease.

The effect of ATRA in this study was mediated by RARα, which induced a transcriptional program that prevented VSMC conversion to an osteogenic phenotype. Diminished ATRA levels have been linked to idiopathic dilated cardiomyopathy (iDCM) in humans, and the authors demonstrated that ATRA supplementation mitigates cardiac remodeling and prevents functional impairment in a guinea-pig model of HF [35]. This finding is consistent with the Bilbija et al. study but suggests that RAR signaling activation may differ between iDCM and MI. 

**Figure 2:**
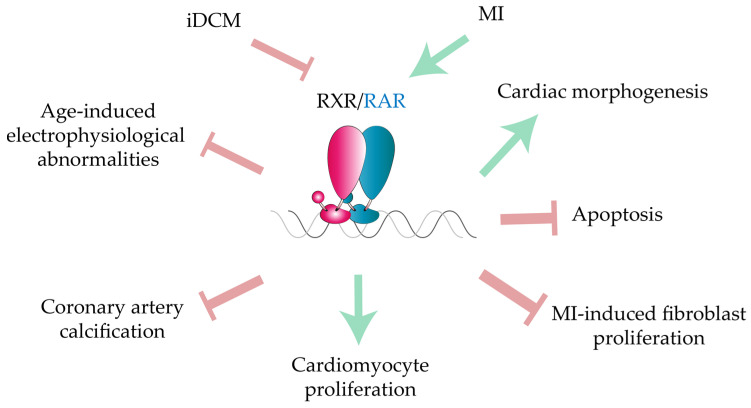
Cardiac-related RARs function. Schematic representation of physiological and pathophysiological cardiac-related processes where RAR/RXR functioning is involved. Regulatory signals for RAR expression are also depicted. Green arrows denote induction. Red arrows denote inhibition. RXR, retinoid X receptor. RAR, retinoic acid receptor. MI, myocardial infarction. iDCM, idiophatic cardiomyopathy.

Together, these studies indicate that ATRA-induced RAR activation can be beneficial in the context of cardiac pathophysiology, but with divergent effects in distinct cell types and pathologies (Figure 2). However, not all of these studies demonstrate a direct effect of the RAR-induced transcriptional program. ATRA protective functions appear to be pleiotropic, affecting several aspects of the pathophysiological response after cardiac injury. Additionally, a variety of effects after ATRA treatment have been reported in rat models of myocardial injury, including reduced inflammation, fibrosis, cardiomyocyte stress, and apoptosis [122,123]. Whether these mechanisms depend on direct RAR activation is unknown, and a role of RXRs cannot be excluded. Notably, one study has linked ATRA cardioprotective functions to the activation of cellular retinoic acid binding protein 1 (Crabp1) [124]. 

Despite the promising scope of exogenous retinoids in clinical use, their teratogenic effects continue to raise substantial concerns. It is, therefore, essential to gain a deep understanding of the specific molecular mechanisms through which RARs mediate cardioprotection, together with more precise studies of the differential involvement of RXRs and RARs in transcriptional pathways during cardiogenesis and CVD.

## 5. Peroxisome Proliferator-Activated Receptors

Peroxisome proliferator-activated receptors are encoded by three distinct genes: PPARα (NR1C1), PPARβ/δ (NR1C2), and PPARγ (NR1C3). The different PPAR isoforms are differentially activated by endogenous unsaturated FAs, and some very low density lipoprotein (vLDL) components and eicosanoids (leukotriene B4 and prostaglandins, including prostacyclin) [125]. In this setting, eicosanoids represent an interesting metabolic signaling hub for PPAR. Their common precursor arachidonic acid, an ω-6 polyunsaturated FA, is differentially oxidized by a complex set of enzymes that give rise to a vast repertoire of bioactive end-products able to modulate gene expression (for review, [126]). Remarkably, fatty acid ethanolamides, naturally generated amides from FA and ethanolamines, have been also highlighted as lipid mediators able to activate PPAR [127,128]. 

PPARs sense dietary lipids, and their main function is the regulation of energy homeostasis by balancing FA consumption and storage in highly metabolic organs [129]. PPARα is widely expressed in liver, muscle, heart, and kidney, PPARδ is ubiquitously expressed, and PPARγ is restricted to adipocytes and some immune cells, such as macrophages [130,131]. 

In recent years, a number of synthetic PPAR ligands have been generated, with fibrates (e.g., fenofibrate, clofibrate, and gemfibrozil) and thiazolidinediones (TZDs:, e.g., rosiglitazone and pioglitazone) used to treat dyslipidemia and diabetes, respectively [125]. All three PPAR isoforms are expressed in cardiac tissue, and their roles have, therefore, been extensively investigated, revealing distinct but crucial regulatory actions required to sustain cardiac homeostasis.

### 5.1. PPARα

PPARα is a master transcriptional orchestrator of cardiac maturation [37]. During the first days of postnatal life, cardiomyocytes undergo a metabolic transition from glucose to mitochondrial FAO as the main energy source. PPARα apparently triggers this fetal-to-neonatal shift by recognizing FAs in maternal milk and initiating the transcription of mitochondrial-related FAO genes [132]. However, mechanistic data confirming the central role of PPARα in perinatal cardiac adaptation are lacking, and further research is needed to determine if PPARα is the only nutrient-sensing effector in this process. Murphy et al. recently showed that regulatory networks driven by PPARα through peroxisome proliferator-activated receptor coactivator-1 (Pgc1α) promote the maturation of pluripotent stem cell-derived cardiomyocytes by regulating YAP1 and SF3B2 proteins [133]. During the fetal-to-neonatal transition, neonatal cardiomyocytes progressively lose their ability to proliferate and enter a hypertrophic phase. Pharmacological and genetic activation of PPARα in infant mice has been shown to transiently sustain cardiomyocyte proliferation and maturation hallmarks such as multinucleation [134]. 

The metabolic function of PPARs in the adult CV system has explored in a variety of mouse models in combination with functional and genome-wide approaches. The first evidence that PPARα is a master regulator of cardiac metabolism was reported by Watanabe et al., who showed that constitutive lack of PPARα blunts cardiac mitochondrial but not peroxisomal FAO by dampening the expression of key enzymes involved in FA catabolism [135]. PPARα-null hearts also show reduced myocardial glucose uptake, suggesting a non-exclusive lipid alteration upon PPARα deletion. PPARα mutant hearts also show cardiac remodeling defects, manifesting as an age-dependent appearance of myocardial fibrosis and abnormal mitochondrial cristae formation. Despite the global defects in cardiomyocyte metabolism in these mice, ATP production in PPARα-null hearts was decreased only in response to food deprivation [135]. In an analysis of the metabolic status of isolated beating PPARα-null hearts, Campbell et al. demonstrated that while palmitate oxidation was reduced, glucose oxidation and glycolysis were increased [136]. The decreased FAO in PPARα-null hearts was associated with increased concentrations of cardiac malonyl-CoA, a potent inhibitor of carnitine palmitoyltransferase I and, therefore, of mitochondrial FA import; these changes were accompanied by decreased expression of malonyl-CoA degradation enzymes but not of glucose transporters. Despite the profound metabolic abnormalities in PPARα-null hearts, survival was not compromised, raising questions about the physiological importance of FAO in the adult myocardium. Dan Kelly’s laboratory demonstrated that treatment of PPARα-null mice with the FAO inhibitor Etomoxir provoked a massive cardiac accumulation of lipids, hypoglycemia, and death [137]. These outcomes showed sex divergence, affecting 100% of males but only 25% of females, suggesting a relationship between PPARs and the hormonal environment. This was further evidenced by the protection against severe outcomes in males after a 2-week pretreatment with b-estradiol. In addition to directly regulating the transcription of FA homeostasis genes, PPARα is also proposed to reduce mitochondrial FAO by modulating the phosphorylation of 5′-adenosine monophosphate-activated protein kinase (AMPK) [138].

**Figure 3:**
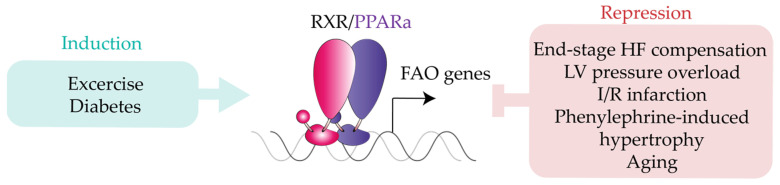
Cardiac stimuli-induced PPAR regulation. Schematic representation of those inductive and repressive signals that modulate cardiac PPAR expression. RXR, retinoid X receptor. PPARa, peroxisome proliferator-activated receptor alpha. FAO, fatty acid oxidation. HF, heart failure. I/R, ischemia/reperfusion. LV, left ventricle.

PPARα activity is modulated by a large amount of cardiac physiological and pathophysiological stimuli (Figure 3). PPARα signaling contributes to the physiological reshaping of glucose and lipid metabolism associated with contraction-induced cardiomyocyte hypertrophy. PPARα protein levels in the hearts of compensated end-stage HF patients are lower than in hearts from healthy donors [139]. In a metabolic analysis of α-adrenergic agonist-induced hypertrophied rodent cardiomyocytes, PPARα dampened palmitate oxidation by decreasing the expression of mitochondrial transporter carnitine palmitoyltransferase I [39]. Analysis of ventricular pressure overload in mice and PPARα overexpression in cardiomyocytes showed that PPARα expression is blunted during pathological hypertrophy, limiting the ability of the myocardium to oxidize lipids [38]. However, cardiac PPARα expression was unchanged in a model of abdominal aortic banding [38], revealing that the regulation of cardiomyocyte hypertrophy is strongly reliant on the stress source. In hypertensive rats treated with the PPARα agonist medium-chain triglyceride tricaprylin, PPARα activation reduced cardiac oxidative stress without affecting blood pressure [140]. More recently, Harvey and colleagues showed that phenylephrine-induced hypertrophy in H9c2 cardiomyocytes reduces PPARα expression while increasing NOX2 expression and activity [141]. In line with this finding, NOX2 expression was upregulated in PPARα-null mice subjected to transverse aortic constriction (TAC), suggesting that NOX2 signaling initiates PPARα downregulation during cardiac hypertrophy. Cardiac hypertrophy can be induced with the widely used β-adrenergic receptor agonist isoproterenol, providing a suitable approach for assessing the therapeutic potential of anti-hypertrophic drugs [142]. Isoproterenol-induced cardiac hypertrophy was significantly attenuated by PPARα activation with fenofibrate or raspberry ketone, as indexed by altered hemodynamic and electrocardiogram patterns and enhanced oxidative stress [143]. Consistent with these findings, Guellich et al. found that mice lacking PPARα had mild systolic dysfunction and dysregulated expression of antioxidative stress enzymes, resulting in impaired myosin motility [144]. Myosin motility alterations and myosin oxidation were reversed by treating PPARα-null mice with a superoxide dismutase mimetic [145]. To investigate the specific relationship between PPARα and reactive oxygen species (ROS) under cardiac stress, Dewald and colleagues administered the PPARα agonist WY-14,643 to mice subjected to repetitive ischemia-reperfusion (I/R) [40]. I/R downregulated the mRNA expression of PPARα, PPARα-dependent FAO enzymes, and myosin heavy chain (MHC). This I/R-induced transcriptional phenotype was prevented by overexpression of superoxide dismutase and aggravated by WY-14,643 treatment, suggesting that PPARα downregulation is an adaptive mechanism to prevent lipotoxicity in the ischemic heart [40]. An examination of the relationship between hypoxia and PPARα signaling revealed decreased cardiac mRNA expression of PPARα and its target genes under hypoxic conditions, although these changes did not affect ATP production or contraction [146]. 

Cardiac PPARα is upregulated in a mouse model streptozotocin-induced diabetes [41]. Moreover, another study showed that PPARα-null mice were protected against DiCM, whereas the disease was worsened in a model of cardiac-restricted PPARα overexpression [147]. PPARα contributed to DiCM in this model by promoting triglyceride accumulation, resulting in a dysregulated oxidative stress status and mitochondrial dysfunction. Notably, Kyriazis et al. recently reported PPARα-independent DiCM progression via molecular crosstalk between FOXO1 and KLF5 [148].

Analysis of the impact of PPARα on atherosclerosis revealed that PPARα-KO on the ApoE-null background surprisingly reduced the number of atherosclerotic lesions, despite an increase in the circulating concentrations of atherogenic lipoproteins [149]. These double-KO mice also showed improved insulin sensitivity and glucose tolerance. Despite this promising metabolic phenotype, the cell-type specific contributions of PPARα to atherosclerosis remain unclear. To explore the myeloid-specific role of PPARα, Babaev et al. reconstituted irradiated low-density lipoprotein receptor–deficient (LDLR^−/−^) mice with either PPARα^−/−^ or PPARα^+/+^ bone marrow [42]. Contrary to the Tordjman study, Babaev et al. found that PPARα deficiency in bone marrow leads to an increase in atherosclerotic lesion size. Accordingly, PPARα^−/−^ peritoneal macrophages strongly induced oxidized LDL uptake and decreased cholesterol efflux, likely due to downregulation of SRB1 and ABCA1 proteins. Collectively, these data suggest that PPARα exerts diverse atherogenic functions depending on the cell context. 

Cardiac expression of PPARα and its FA metabolism target genes decreases with age but increases with exercise [150], which suggests that myocardial PPARα signaling might improve age-dependent FA use. Exercise has also been linked to the protective action of PPARα in MI [151]. These authors found that exercised rats subjected to MI had better cardiac function and lower levels of inflammatory markers and that these variables correlated with higher PPARα expression than in the sedentary group. In a separate approach, inflammation and infarct size in rats were decreased upon PPAR-signaling activation with clofibrate [152]. In this analysis, clofibrate treatment blocked the induction of cardiac remodeling signals such as oxidative stress pathways, suggesting that PPARα is an important coordinator of myocardial stress signaling. 

### 5.2. PPARδ

The pivotal role of PPARδ in cardiovascular physiology was established by Cheng et al., who reported this isoform as the predominant subtype in the heart [43]. Cardiomyocyte-specific deletion of PPARδ (CM-PPARδ) results in a downregulation of key FAO genes that limits the myocardial lipid-consumption rate, leading to triacylglyceride accumulation, cardiac hypertrophy, congestive HF, and reduced survival [44]. These findings identified PPARδ as an essential regulator of FA homeostasis and cardiac energetics. In response to fasting or a high-fat diet, CM-PPARδ hearts increase the transcript expression of FAO machinery genes without increasing protein levels [153], suggesting that PPARδ is essential for myocardial FAO maintenance. Notably, cardiac PPARδ deficiency correlated inversely with ROS production, cell hypertrophy, and protein synthesis in H9c2 embryonic rat cardiomyocytes exposed to hyperglycemia, suggesting a possible role in DiCM [53]. In line with this finding, PPARδ deficiency and associated lipid deposition in diabetic hearts are counteracted by digoxin, possibly through partial rescue of FAO activity [154].

Pharmacological activation of PPARδ with the agonist GW1516 has been shown to attenuate atherosclerotic lesion development by reducing aortic sinus lesions and the number of myeloid cells within the plaque, promoting transition from the M1-like to the M2-like macrophage phenotype [46]. This atheroprotective effect was also reported by Takata and colleagues, who showed that the PPARδ agonist GW0742 attenuated AngII-accelerated atherosclerosis regardless of blood-pressure changes [47]. This effect was mediated by PPARδ-induced expression of anti-inflammatory genes such as Bcl-6, together with inhibition of AngII-activated MAPK, p38, and ERK1/2. The impact of PPARδ activation has also been evaluated in relation to AngII-induced cardiac hypertrophy, revealing an ameliorating effect of the agonist GW0742 in neonatal rat cardiomyocytes [50]. GW0742 treatment reduced cardiomyocyte expression of both matrix remodelers and inflammatory mediators, demonstrating a multi-faceted protective role of PPARδ in cardiac physiology.

PPARδ signaling activation has been linked to protection against oxidative stress. The selective PPARδ agonist GW501516 protects H9c2 cardiomyocytes from H_2_O_2_-induced cell death by upregulating enzymatic antioxidative defenses [155]. Increased PPARδ expression has been linked to the key inflammatory mediator C-reactive protein (CRP), and CRP-induced NF-kB and IL-6 expression in cardiomyocytes and hypoxia-induced cell apoptosis in H9c2 cardiomyoblasts were blocked with the synthetic PPARδ agonist L-165041 [156]. L-165041 also blocks LPS-induced NF-kB activation and prevents phenylephrine-induced hypertrophy in H9c2 cells by maintaining the levels of FAO-related enzymes [157].

MI is accompanied by a marked dysregulation of the inflammatory response that provokes necrosis and massive cell damage [158]. Chronic treatment of MI-induced rats with the synthetic PPARδ activator GW610742X normalized FAO rates in a dose-dependent manner, and treated mice showed a sharp reduction in cell hypertrophy in the right ventricle and a reduced rate lung congestion, suggesting PPARδ intervention as a plausible therapeutic approach for HF [159].

### 5.3. PPARγ

The inflammation-regulatory action of PPARγ has provoked interest in its impact on myocardial injury [160]. PPARγ ligands have been reported to ameliorate MI through anti-inflammatory effects [161,162,163], and cardiomyocyte-specific PPARγ deletion results in extensive myocardial damage, characterized by increased ventricular dilation, neutrophil infiltration, and elevated proinflammatory cytokine concentrations [164]. Additionally, myeloid-specific PPARγ in a mouse model of MI resulted in increased infarct size, enhanced cardiac hypertrophy, and greater expression of injury markers such as natriuretic peptide b (*Bnp*) and the oxidative stress enzymes *Nox2* and *Nox4*, revealing a protective mechanism through which macrophage-expressed PPARγ limits the impact of MI [49]. In an in vitro model of I/R injury, cardiomyocytes showed strong downregulation of PPARγ and KLF5 expression, whereas Pgc1α was induced [165]. In this model, overexpression of KLF5 prevented the expression of inflammatory mediators and diminished apoptosis by enhancing PPARγ expression during injury. These findings establish the existence of a novel KLF5–PPARγ–Pgc1a pathway that protect cardiomyocytes against I/R injury. Moreover, PPARγ-dependent anti-inflammatory mechanisms and NF-kB pathway repression are implicated in the simvastatin-mediated prevention of cardiopulmonary bypass-induced inflammation [166]. PPARγ activity is also implicated in the anti-inflammatory mechanisms activated by curcumin [167], and oral administration of curcumin to SH rats attenuated cardiac fibrosis and dysfunction by increasing PPARγ expression in ventricular cardiomyocytes [168]. Moreover, curcumin treatment of cardiac fibroblasts blocked the AngII-induced production of extracellular matrix by inhibiting TGFβ1 expression and Smad2/3 phosphorylation in a PPARγ-dependent manner, further supporting the cardioprotective role of this NR. 

The role of PPARγ has been also explored in relation to DiCM. The selective PPARγ agonist rosiglitazone is widely used to treat type 2 diabetes; however, this treatment is associated with an increased risk of MI and CV death [169]. Pioglitazone, another selective PPARγ agonist, was recently demonstrated to ameliorate type 1 DiCM in diabetic Sprague Dawley rats by depressing the Ca^+2^/calmodulin-dependent protein kinase II (CaMKII) pathway, correlating with reductions in oxidative stress and cardiac fibrosis [52]. Pioglitazone cardioprotection has also been observed in diabetic mice, revealing an alternative mechanism by which PTEN/AKT/FAK modulation reduces collagen deposition and pathological DiCM hypertrophy [51]. In contrast, the tesaglitazar and the glitazar classes of dual PPARα and PPARγ agonists has been shown to worsen cardiac function in type 2 diabetes patients [170,171]. 

PPARγ agonists also show beneficial effects in atherosclerosis [172]. For example, the PPARγ activator troglitazone has been shown to inhibit monocyte/macrophage recruitment to the atherosclerotic plaque in ApoE-null mice. Cultured ECs treated with troglitazone downregulated *Vcam1* and *Icam1* transcription, key players in vascular inflammation [173]. Furthermore, Chris Glass’s laboratory showed that the PPARγ-specific agonists rosiglitazone and GW7845 have anti-atherogenic effects, improve insulin sensitivity, and diminish local and systemic expression of inflammatory markers in LDLR^−/−^ mice [48]. The intricate protective role of PPARγ in diabetes and atherogenesis opens a promising therapeutic approach towards the treatment of CV risk associated with diabetes. 

Interestingly, vascular PPARγ deletion provokes circadian rhythm alterations and heart rate abnormalities, and PPARγ has a robustly rhythmic expression pattern in the aorta [174]. The same study showed that PPARγ directly targets the canonical clock gene Bmal1, establishing a role for this NR in determining cardiovascular rhythms. 

**Figure 4:**
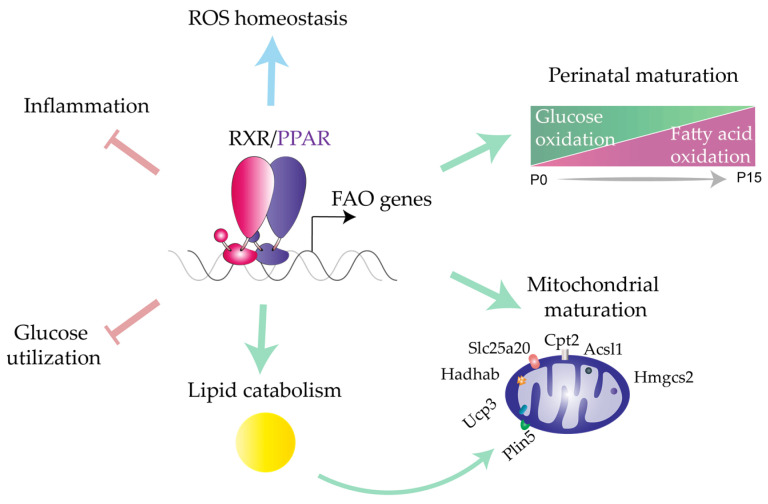
Cardiac-related PPARs function. Schematic representation of physiological cardiac-related processes where PPAR/RXR functioning is involved. Blue arrows denote homeostatic regulation. Green arrows denote induction. Red arrows denote inhibition. RXR, retinoid X receptor. PPAR, peroxisome proliferator-activated receptor. FAO, fatty acid oxidation. ROS, reactive oxygen species. P0, postnatal day 0. P15, postnatal day 15. Some PPAR mitochondrial target genes are depicted: Ucp3 (Uncoupling protein 4), Plin (Perilipin 5), Hmgcs2 (3-Hydroxy-3-Methylglutaryl-CoA Synthase 2), Acsl1 (Acyl-CoA Synthetase Long Chain Family Member 1), Cpt2 (Carnitine Palmitoyltransferase 2), Slc25a20 (Solute Carrier Family 25 Member 20), Hadha/b (Hydroxyacyl-CoA Dehydrogenase Trifunctional Multienzyme Complex Subunit Alpha and Beta).

Collectively, the dynamic involvement of PPAR in CV physiology and its distinct roles in response to cardiac insult signals (Figure 4) the need for a deeper molecular characterization of the therapeutic potential of PPAR agonists.

## 6. Liver X Receptors

Liver X receptors, although first identified as orphan receptors, are able to respond to oxysterols and act as cholesterol sensors [175]. Oxysterols comprise a family of 27-carbon cholesterol oxidized derivatives or by-products of cholesterol biosynthesis (for review [176]). They are mainly generated during the metabolic conversion of cholesterol to bile acids or steroid hormones by the cytochrome P450 family enzymes (CYP). Among them, CYP27A1 and CYP46A1 isoforms represent the most important cholesterol hydroxylases for LXR signaling pathway. 

Two LXR isoforms were discovered in the 1990s by separate laboratories, with each isoform showing a distinct pattern of expression. LXRα (NR1H3) [177] is restricted to tissues with a high lipid metabolic turnover such as the liver, whereas LXRβ (NR1H2) [178] is expressed almost ubiquitously.

LXR target genes are crucial components of cholesterol metabolism, and LXRs regulate cholesterol homeostasis through a variety of processes such as cholesterol absorption, uptake, conversion, and transport. This is achieved via an interconnected and tissue-specific modulation of transcription in the intestine, liver, and peripheral tissues. LXRs are believed to play a role in metabolic syndrome and its constituent disorders (obesity, type 2 diabetes, etc.). In relation to cardiovascular physiology, most studies to date have focused on systemic effects, but LXRs may also directly regulate the heart through the strong relationship between cardiac homeostasis and metabolism.

LXRs have been postulated to regulate hypertension, one of the most prevalent co-morbidities in CVD patients and a major risk factor for atherosclerosis, HF, and hypertrophy. LXRs are thought to regulate hypertension by inhibiting the renin-angiotensin-aldosterone system (RAAS) and by increasing the expression of cardiac natriuretic peptides [179]. The use of LXR agonists in pressure-overload models indicates that LXR activation can alleviate the consequences of induced hypertension in rodents [180,181,182], suggesting the potential of LXRs as a therapeutic target. However, it remains unclear exact what molecular mechanisms underlie these observations and whether they depend on an LXR-dependent transcriptional signature.

Research into the anti-atherogenic action of LXRs has revealed an action through the modulation of cholesterol metabolism not only in the liver but throughout the body [183,184]. Bone marrow transplantation experiments revealed that LXRs can prevent atherosclerosis through their actions in macrophages and other hematopoietic cells [185,186]. The synthetic LXR ligand GW3965 has been shown to have an anti-atherogenic effect when administered to ApoE-null mice [187]. In macrophages, LXRs reduce foam-cell formation by promoting cholesterol efflux and modulate their transcriptional status towards a M2-like phenotype [55]. Interestingly, LXRs also appear to provide protection against atherosclerosis through an anti-inflammatory action in endothelial cells (EC) [56,188] and by promoting cholesterol efflux from aortic SMCs [189].

The heart expresses both LXR isoforms, but the highest expression levels correspond not to cardiomyocytes, but to ECs and fibroblasts [190]. An involvement of LXRs in cardiac pathophysiology is further supported by the induction of LXR expression in cardiac conditions such as MI, I/R injury, and DiCM [179,191].

Myocardial I/R injury is increased in LXR-deficient mice and reduced in LXR-overexpressing mice, with the cardioprotection mediated by LXRα, but not by LXRβ, through a reduction in cardiomyocyte apoptosis induced by ER stress and mitochondrial oxidative/nitrosative stress [57]. LXR activation also promotes cell survival after MI through the TLR4/NFκB and Keap-1/Nrf-2 signaling pathways [192]. Interestingly, a more recent study revealed sex differences in the post-MI response associated with LXR-RXR signaling in neutrophils [193]. In this study, old female mice did not depend on LXR signaling for recovery after MI, an important finding given the current interest in LXRs as a promising therapeutic target in CVD.

LXRα-induced reduction of oxidative/nitrosative stress also provides protection against DiCM [58]. A separate study showed that LXRα mediates crucial cardiac metabolic changes that improve heart function in a mouse model of obesity-induced type 2 diabetes [60]. These authors proposed natriuretic peptides *Anf* and *Bnp* as potential direct LXRα target genes, suggesting that LXRα contributes to the initiation of the cardiac stress response as a mechanism to promote cardioprotection. The cardioprotective effects of LXRs in the context glucose-induced injury have been explored in in vitro studies, revealing that LXRα is regulated by a miRNA and reduces apoptosis through the endogenous mitochondrial pathway [59,194]. Another report described mediation of LXRα regulation by nuclear receptor corepressor (NCoR) via the transrepression of target genes related to apoptosis and inflammation, providing protection against cardiomyocyte stress [195].

Given the ability of cardiomyocytes to remodel metabolic networks [132,196], the crucial metabolic modulator function of LXRs suggests that they could regulate cardiomyocyte pathophysiological responses. LXRα cardioprotection has been proposed to operate through an increase in glucose uptake and use [179]. The same group previously showed that cardiac LXRα protects against myocardial hypertrophy and DiCM by inducing *Glut1* and *Glut4*, thus promoting increased glucose uptake and metabolism [60,61]. In a rat diabetes model, the synthetic LXR agonist TO901317 reduced diacylglycerol accumulation in the heart, a hallmark of DiCM, regardless of differential expression of LXR isoforms [62]. This also suggests that LXRs might contribute to the regulation of alternative metabolic mechanisms in the injured heart, consistent with the idea that rewiring myocardial metabolism to promote glucose utilization in the heart correlates with better post-injury outcomes [197,198]. 

LXRα is also upregulated during cardiac hypertrophy [179,191]. LXR activation reduces cardiac hypertrophy in several models, including TAC in mice [199] and pulmonary artery hypertension (PAH) in rats [200]. In these models, the hypertrophy-limiting effect of LXRα was mediated by the inhibition of the TGFβ and NFκB inflammatory pathways, respectively. A more recent study revealed upregulation of the LXR-RXR pathway in samples of human hypertrophic cardiomyopathy, suggesting that LXRs also play an important role in cardiac disease in humans [201]; however, whether this effect contributes to cardioprotection is unknown. Strikingly, a pig model of pacing-induced cardiomyopathy revealed inhibition of the LXR-transcriptional pathway accompanied by increased hypertrophy and fibrosis [202]. This result hints at a complex upstream regulation of LXRs, which may be different in the context of pacing-induced cardiomyopathy.

In the heart, only LXRα is upregulated upon cardiac injury, and it is the predominant isoform mediating protective functions, mostly by reducing cardiomyocyte oxidative stress, inflammation, and apoptosis. Nevertheless, uncertainty remains about whether the LXR cardioprotective effects depend on decreasing insulin resistance or on modulation of the balance between glucose and lipid metabolism, and a cell-autonomous effect of LXRα cannot be ruled out with the current evidence. Further studies are, therefore, required using more refined animal models and state-of-the-art techniques.

Cardiac remodeling through inflammation and fibrosis is also a key player in myocardial I/R injury, hypertrophy, and DiCM. Moreover, LXRs may perform protective functions through actions in other cardiac cells, such as fibroblasts and macrophages, which are enriched in the main signaling pathways and functional processes in which LXR is implicated. The evidence suggests that LXRs modulate the AngII, TGFβ, and NFκB pathways in cardiac fibroblasts and macrophages [179], supporting the idea that LXRs are pleiotropic effectors in the heart. 

**Figure 5:**
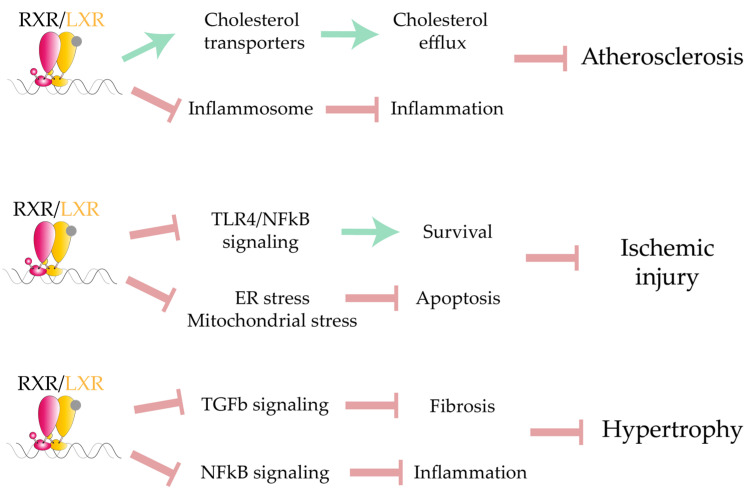
LXR function during CVD. Schematic representation of cardiac pathophysiological processes where LXR/RXR functioning is involved. Green arrows denote induction. Red arrows denote inhibition. RXR, retinoid X receptor. LXR, liver X receptor. TLR4, Toll-like receptor 4. NFkB, nuclear factor kappa light chain enhancer of activated B cells. TGFb, transforming growth factor-Beta. ER, endoplasmic reticulum.

Current knowledge indicates that LXRs are major regulators of homeostatic pathways related to cardiovascular physiology, providing protection against CVD through a complex network involving the interaction of multiple tissues and cell types (Figure 5). While the molecular mechanisms driven by LXRs in liver and macrophages are well characterized, the precise transcriptional response underlying the cardioprotective effects of LXRs has not been clearly defined. Targeting LXRα specifically in the heart is an attractive candidate for an effective therapeutic strategy; however, progress in this direction will require a full understanding of transcriptional regulation upstream and downstream of LXRs, the LXR co-regulatory complexes in this tissue, and an intense drug discovery program. 

## 7. Thyroid Hormone Receptor

Thyroid hormone receptors respond to the hormones 3,5,3′-triiodothyronine (T3) and 3,5,3′,5′-tetraiodothyronine (T4, thyroxine), which are endogenously produced in the thyroid glands [203]. Although both thyroid hormones (TH) are biologically active, T3 is the more potent ligand [203,204]. In humans, but not mice, T3 can be produced by T4 deoinization through the action of seleno cysteine enzymes called deiodinases [204,205,206]. Thyroid hormone receptors (TR) are encoded by two genes, TRα (NR1A1) and TRβ (NR1A2), both of which generate several splice variants [207]. These genes show differential expression throughout the body, and TRs are detected in the myocardium [208] and the vasculature [209], evidencing a functional niche for TH signaling in the CV system. In the mouse heart, the predominant isoforms are TRα1 and TRβ1, respectively accounting for 70% and 30% of TR mRNA abundance [210,211]. Although TRs can function as monomers or homodimers, the highest affinity for DNA response elements is seen with TR/RXR heterodimers, which induce a global transactivation of TR-dependent genes [208]. 

Circulating TH concentrations are intimately linked to cardiac function, impacting cell status through two modes of action: genomic, involving binding to TRs in cell nuclei, and non-genomic, involving direct modulation of the peripheral circulation and thereby cardiac filling, contractility, CV hemodynamics, and ion channel permeability in the cardiomyocyte membrane [212,213]. Here, we focus on the genomic actions of TRs.

TR signaling contributes to the regulation of cardiac contraction. Hyperthyroid patients present a wide variety of CV alterations, including palpitations, widened pulse pressure with systolic hypertension, and low diastolic pressure [214,215]. The increased cardiac output in these patients results from the combination of peripheral vascular effects and the cell-autonomous enhancement of myocardial contractility. In contrast, subclinical hypothyroidism is related to diastolic dysfunction and impaired contractility, placing these patients at an elevated risk of MI and atherosclerosis [216]. 

**Figure 6:**
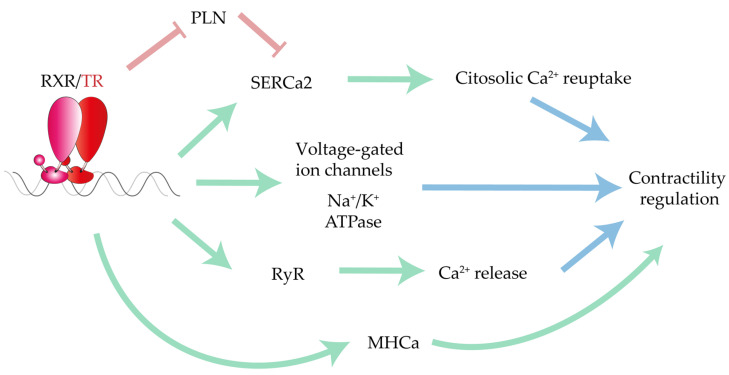
TR signaling in cardiomyocyte contractility. Schematic representation of TR/RXR functioning during cardiac contraction. Blue arrows denote homeostatic regulation. Green arrows denote induction. Red arrows denote inhibition. RXR, retinoid X receptor. TR, thyroid hormone receptor. SERCa2, Sarcoplasmic/Endoplasmic Reticulum Calcium ATPase. PLN, Phospholamban. RyR, Ryanodine Receptor.

One of the most important T3-responsive genes is the calcium pump located in the sarcoplasmic reticulum, SERCa2, which is in charge of lowering cytosolic calcium during diastole [217]. Calcium release and reuptake paces systolic contractile function and heart rate. TH concentrations also influence the SERCa2 regulator phospholamban (PLN) [218], with PLN expression depressed in hyperthyroid hearts but increased in hypothyroid hearts [219]. Accordingly, T3 treatment of rat ventricular cardiomyocytes increased the SERCa2:PLN ratio and decreased PLN protein content, enhancing contraction and the speed of calcium transient currents [65]. In addition to controlling SERCa2-dependent calcium reuptake, T3 also increases ryanodine-mediated (RyR) calcium release to the cytosol during systole by directly increasing RyR mRNA expression [66]. The positive inotropic effect of T3-TR signaling are further evidenced in studies by Gick et al. and Ojamaa et al., who reported that mRNA expression of the key ion transporters voltage-gated K^+^ channel (Kv1.5) and Na^+^/K^+^ ATPase are upregulated in contracting hyperthyroid hearts and cultured myocytes, respectively (Figure 6) [64,67].

In the myocardium, T3 regulates the expression of myosin heavy chain (MHC) α and β, which are key sarcomere components of the myofibrillar contractile apparatus in mammalian cardiomyocytes [220]. In vivo and in vitro approaches have shown that T3 increases expression of MHCα while repressing MHCβ [221,222]. Analysis of the T3-TR cistrome identified positive and negative regulatory sequences in the MHCα and MHCβ promoters, respectively [222]. Hypothyroidism in rats also diminishes the expression of cardiac troponin I, a structural component of thin filaments [223]. The overall effect of T3-TR signaling in cardiomyocytes is thus a transactivation of contractile and inotropic genetic programs, resulting in an increase in protein synthesis that impacts cell size [224]. This is also evidenced in the increased expression of the hypertrophy markers *Anf* and *Bnp* [224,225]. MHC and SERCa2 mRNA expression are also regulated by exercise training, which can prevent aging-induced downregulation of these genes. Iemitsu et al. demonstrated that TRα1 and TRβ1 mRNA abundance declines with age but increased when rats were placed on a physical activity program [226]. Furthermore, SERCa2 and MHC protein content correlated directly with TR expression, resulting in improved cardiac function in the trained rat group and indicating that TRs orchestrate exercise-derived functional adaptations in the heart. 

Several loss-of-function mouse models have been generated to explore the roles of T3-TR signaling in the CV system. Fraichard and colleagues generated the first homozygous deletion of TRα by targeting exon 2 (TRα^−/−^), producing mice that died 5 weeks after birth [227] and had reduced body size and bradycardia at baseline and under TH stimulation [228]. A second TRα mutant mouse line (TRα^0/0^) was generated by deleting exons 5–7; these mice reproduced the heart rate abnormalities of TRα^−/−^ mice but were viable [229]. Moreover, double TRα^0/0^/TRβ mutant mice presented more severe growth retardation without any impact on survival [229]. Both TRα^−/−^ and TRα^0/0^ atrial and ventricular cardiomyocytes showed diminished expression of the cyclic nucleotide-gated ion channels HCN2 and HCN4 [211] and the *Serca2* and *Myhcα* genes [230]. These transcriptional alterations likely underlie the inotropic and contractile phenotypes in these mutants. In contrast to the cardiac phenotype of TRα-deficient mice, systemic deletion of the *TRβ* gene (TRβ^−/−^) provoked an 11% increase in basal heart rate, although cardiac function was preserved [231]. The differential expression pattern of ion channel and contractile genes was not reported in this model. Simultaneous deletion of TRα and TRβ resulted in abnormal electrophysiological parameters, which again caused bradycardia and impaired contractile function [210].

TRα1 transcript abundance is downregulated in a setting of post ischemic cardiac dysfunction [232]. In this setting, TH treatment has been widely shown to ameliorate MI-induced cardiac damage. Chen et al., demonstrated that T3 administration prevents cardiomyocyte apoptosis via Akt signaling, improving left ventricle dilation upon injury [69]. Another study performed by Pantos and colleagues, reported an alternative mechanism by which TH treatment reversed cardiac dysfunction by regulating cardiomyocyte geometry and myosin isoforms expression [68]. Collectively, these findings indicate that TRα is the predominant isoform implicated in the modulation of heart homeostasis; however, further research is needed to delineate the potential of treatments targeting TR signaling in CVD.

## 8. Vitamin D Receptor

Vitamin D receptor (VDR) is a nuclear hormone receptor whose endogenous ligand is the bioactive metabolite of vitamin D, 1,25 vitamin D3 (1,25(OH)_2_D3) [233]. Although its main physiological functions are related to mineral and bone homeostasis, growing evidence suggests a functional impact beyond calcium and phosphate metabolism [234,235,236,237,238]. In humans, VDR is encoded by the NR1I1, gene and its presence has been detected throughout the body, including immune cells, intestine, and the renal system [239]. Several studies have reported that cardiac tissue expresses not only VDR but also 1α- and 24α-hydroxylases, the enzymes responsible for the endogenous synthesis of 1,25(OH)_2_D3 [240,241,242]. The presence of the complete vitamin D signaling pathway machinery in the heart suggests of hints for a physiological role of this NR in cardiovascular homeostasis. 

Vitamin D deficiency is intimately linked to the development of CVD [241,243]. Liu et al. found that low 1,25(OH)_2_D3 concentrations in plasma correlated with poor prognosis in HF patients and a higher mortality risk. In the same study, increased abundance of *pro-Bnp* was inversely correlated with serum vitamin D concentration, indicating sustained RAAS activation [243]. The patients in this study also had an elevated abundance of pro-inflammatory markers [243]. RAAS signaling is highly dysfunctional in chronic kidney disease patients, who frequently develop cardiac conditions, strengthening the case for interplay between RAAS and VDR biology as a possible orchestrator of vitamin D-derived HF [244]. Further evidence for a relationship between vitamin D status and cardiac homeostasis comes from the myocardial dysfunction reported in infants with severe hypocalcemia [245,246,247]. Collectively, these epidemiological studies demonstrate a protective role of vitamin D in the maintenance of cardiac function in humans, while suggesting the existence of intricate cell-specific mechanisms that were not completely understood. 

**Figure 7:**
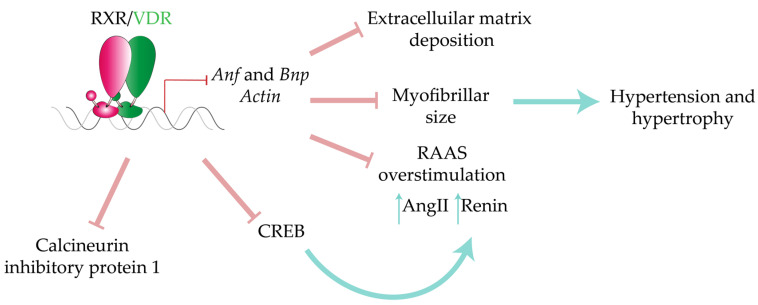
VDR anti-hypertrophic regulatory functions. Schematic representation of VDR/RXR modulation during pathological hypertrophy. Green arrows denote induction. Red arrows denote inhibition. RXR, retinoid X receptor. VDR, vitamin D receptor. RAAS, renin-angiotensin system. CREB, cAMP response element-binding. AngII, angiotensin II. Anf, atrial natriuretic peptide. Bnp, brain natruiretic peptide.

The VDR signaling pathway has been shown to exert antihypertrophic functions in cardiac cells. In vitro studies have demonstrated that 1,25(OH)_2_D3 administration prevents the expression of the hypertrophy markers *Bnp* and *Anf* and of alpha-skeletal actin in primary rat cardiomyocytes [248,249] and cultured HL-1 myocytes [242]. Accordingly, nutritional vitamin D depletion in Sprague Dawley rats resulted in an increased heart-weight/body-weight ratio, a smaller myofibrillar area, and increased extracellular space [250]. The hypertrophic features in vitamin D-deficient female rats were recapitulated in their offspring, in which cardiomyocyte size and number were also significantly increased [73]. The Gardner group reported the upregulation of VDR mRNA and protein in hypertrophied myocytes from isoproterenol-induced Wistar rats [74]. Consistent with these results, cardiac hypertrophy and impaired systolic function were reported in systemic and cardio-specific VDR-KO mice [71,72,75]. VDR deletion also resulted in detrimental cardiac contraction in response to isoproterenol [75]. Importantly, hypertension, cardiac hypertrophy, and contractility alterations are also detected in systemic 1α-hydroxylase KO mice [251]. Collectively, these results establish the protective role of VDR and 1,25(OH)_2_D3 in cardiac homeostasis and hypertrophy (Figure 7). 

To define the molecular basis of VDR regulatory mechanisms in the CV system, it is important to distinguish between the non-cell autonomous and cell-autonomous contributions of this NR to cardiac hypertrophy. Systemic VDR disruption results in RAAS overstimulation, with a concomitant increase in renin and AngII in the kidneys that indirectly causes cardiac hypertrophy [72]. A similar renin upregulation was observed in myocytes from cardiac-specific VDR-KO mice [75]. The mechanism of renin repression by the active VDR is thought to involve interaction with cyclic AMP response element binding protein (CREB), which drives renin promoter activation [252]. In addition, cardiac-specific VDR-KO has been shown to directly regulate *Bnp* promoter activity [74]. Accordingly, in vitro treatment of neonatal cardiomyocytes with 1,25(OH)_2_D3 decreased the expression of modulatory calcineurin inhibitory protein 1 [75]. The same treatment in HL-1 myocytes reduced cell proliferation and *Anf* expression [242]. Hypertension, upregulated RAAS activity, and cardiac hypertrophy and dysfunction are also produced upon systemic deletion of 1α-hydroxylase, which prevents the endogenous synthesis of bioactive vitamin D [251]. The blood pressure, RAAS, and cardiac alterations in 1α-hydroxylase KO mice were reverted by treatment with 1,25(OH)2D3, demonstrating that the underlying mechanism was vitamin D-dependent [251]. Given the key role of hypertension signaling in hypertrophy, several studies have explored the therapeutic potential of targeting the RAAS axis. The widely used anti-hypertensive drugs captopril and losartan reversed the altered cardiac structure and function in the systemic VDR and 1α-hydroxylase KO models [251,253], suggesting novel molecular mechanisms to target for the development of new pharmacological strategies.

VDR activity also contributes to cardiac fibrosis and extracellular matrix remodeling through the regulation of the expression of ECM mediators such as matrix metalloproteinases (MMP) and tissue inhibitors of metalloproteinases (TIMP) [254]. Lack of VDR induces myocardial expression of MMP-2 and MMP9 and dampens myocardial TIMP-1 and TIMP-3 expression, correlating with cellular hypertrophy and cardiac fibrosis [76]. Tests of the anti-fibrotic effect of VDR in humans revealed that vitamin D-deficient individuals have significantly increased blood concentrations of TIMP-1 and MMP-9 [255]. Vitamin D deficiency also correlates with a worse phenotype after MI [256,257,258]. The infarcted human heart is unable to regenerate, and efforts are, therefore, increasingly aimed at deciphering the complex molecular mechanisms underlying cardiomyocyte proliferation. Han et al. describe that pharmacological or genetic manipulation of vitamin D signaling controls cardiomyocyte proliferation in zebrafish hearts [259]. Notably, systemic VDR-KO mice showed a significant increase in HF biomarkers, apoptosis, inflammation, and fibrosis, indicating that vitamin D plays a protective role during MI [260]. 

Other groups have explored whether VDR plays a beneficial role in atherosclerosis. Systemic deletion of VDR on the LDLR^−/−^ background induces atherogenesis, increases aortic levels of proinflammatory cytokines, and enhances foam-cell formation [78]. In line with this finding, LDLR^−/−^ and ApoE-null mice first fed a vitamin D deficient diet and then a high-fat diet develop hypertension and higher plasma renin and numbers of atherosclerotic plaques than those fed a vitamin D sufficient diet [79]. Interestingly, macrophages from the vitamin D deficient group had a marked M2 phenotype, ER stress, and elevated foam-cell formation [79]. Oral treatment with calcitriol, an active form of vitamin D, inhibits atherosclerosis development by modulating dendritic-cell differentiation and regulatory T cell function [80]. This finding evidences the complex and pleiotropic roles of VDR in atherosclerosis.

## 9. Nur77

Nur77 (also known as NGFI-B) is encoded by the NR41 gene, an immediate–early response NR whose expression depends on a vast variety of extracellular stimuli, including growth factors, cyclic-AMP-dependent pathways, cytokines, and hormones [261]. To date, no endogenous ligand has been identified, and Nur77 is considered an active transcriptional unit that functions in a ligand-independent manner. 

**Figure 8:**
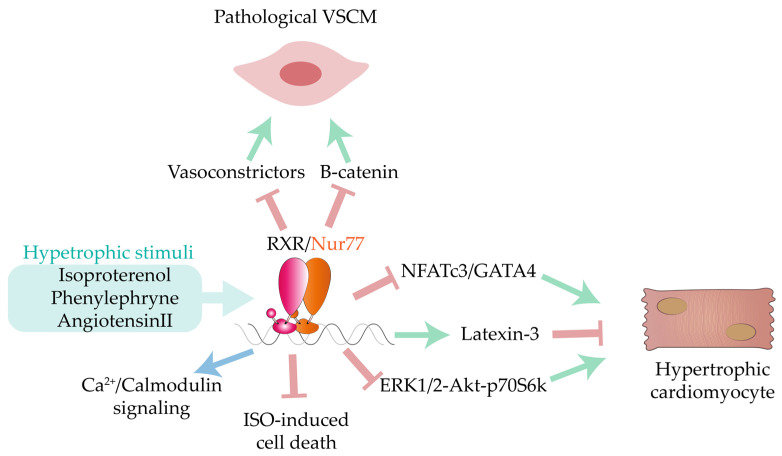
Nur77 anti-hypertrophic functions. Schematic representation of Nur77/RXR modulation during pathological hypertrophy. Inductive signals for Nur77 in this setting are also depicted. Green arrows denote induction. Red arrows denote inhibition. Blue arrows denote homeostatic regulation. RXR, retinoid X receptor. ISO, isoproterenol. ERK1/2, extracellular signal-regulated kinase 1/2. Akt, Protein kinase B. p70S6K, Ribosomal protein S6 kinase beta-1. NFATc3, Nuclear Factor of Activated T Cells 3. GATA4, Transcription Factor GATA-4.

Cardiomyocyte expression of Nur77 mRNA is induced by hypertrophic stimuli such as isoproterenol and phenylephrine [82] (Figure 8). Consistent with this finding, cardiomyocyte hypertrophy is induced by Nur77 knockdown in vitro, and Nur77-null mice have abnormal action potentials and Ca^2+^/calmodulin signaling, resulting in enlarged cardiomyocytes and fibrosis upon isoproterenol stimulation [84]. In contrast, Nur77 deficiency protected against TAC-induced cardiac hypertrophy. In agreement with the Medzikovic et al. study, cardiac-overexpressed Nur77 inhibited isoproterenol-induced hypertrophy by interacting with NFATc3/GATA4 and blocking its nuclear action [82]. In a separate study, Ashraf et al. reported that Nur77 overexpression in isoproterenol-treated ventricular rat cardiomyocytes blocks the phosphorylative activation of the growth-related kinases ERK1/2, Akt, and p70 S6 kinase [83]. In this case, the direct transcriptional effects of Nur77 were mediated by the inhibitory action of dual-specificity phosphatases 1 and 2 on ERK1/2, resulting in diminished hypertrophic cell growth. Nur77 was also shown to induce the expression of latexin-3 peptide, a member of relaxin family, whose members exert beneficial anti-apoptotic, anti-fibrotic, and anti-hypertrophic effects in the CV system [85,86]. In these experiments, isoproterenol increased latexin-3 mRNA expression in a Nur77-dependent manner, prompting examination of the impact of Nur77 overexpression on the pro-apoptotic effects of isoproterenol. Nur77 overexpression significantly reduced isoproterenol-induced cell death, suggesting a cardioprotective action of latexin-3/Nur77 signaling [85]. Interestingly, serum from Nur77-null mice provoked a severe hypertrophy in cultured neonatal rat cardiomyocytes that was inhibited by the neuropeptide Y type I receptor antagonist BIBO3304, evidencing another promising mechanism through which Nur77 might be targeted in HF [18].

Nur77 also exerts pleiotropic effects on the complex signaling in ECs that determines vascular tone through the balance between vasodilation and vasoconstriction, a key determinant in the development of CVD. AngII induces Nur77 expression in cultured rat VSMCs [262], and Nur77 is also increased in aortic VSCMs from AngII-infused mice [88]. Nur77 has been proposed to negatively regulate AngII-induced VSCM proliferation by inhibiting β-catenin activity [88], and ectopic Nur77 expression or pharmacological activation with 6-mercaptopurine (6-MP) inhibited the expression of the potent vasoconstrictor endothelin-1 (ET1), produced by vascular ECs, through negative regulation of c-Jun/AP-1 signaling [81]. Consistently, in vitro knockdown of Nur77 in ECs and aort from Nur77-null mice increased thrombin-stimulated ET1 expression. A recent study showed that Nur77, together with Nor1, coordinates human EC expression of thrombomodulin, a key anticoagulant and anti-inflammatory mediator [87]. EC apoptosis and mitochondrial damage in a model of cardiac microvascular I/R injury is associated with marked upregulation of Nur77 transcript expression, and this EC injury was not seen after I/R in Nur77-null mice [89]. In this setting, the authors propose that Nur77 triggers overactivation of the serine/threonine kinase casein kinase2 α, resulting in increased mitochondrial fission and ultimately triggering endothelial apoptosis and microvascular dysfunction. Together, these studies establish Nur77 as an attractive pharmacological target for the treatment of microvascular injury, arterial thrombosis, and hypertension-induced CVD.

Deletion of Nur77 on the LDLR^−/−^ or ApoE^−/−^ genetic backgrounds worsens high-fat-diet–induced atherosclerosis, indicating an involvement of Nur77 in atherogenesis [263]. The effect of Nur77 deletion in these mice was associated with substantial macrophage polarization toward a pro-inflammatory phenotype, and recent work by Koenis and colleagues showed that Nur77 acts as a critical upstream regulator of the pro-inflammatory metabolic switch in macrophages, which is led mainly by changes to the α-ketoglutarate:succinate ratio [90]. Nur77 deficiency triggers an abnormal metabolic rewiring in macrophages that leads to an exacerbated inflammatory response that contributes to atherosclerosis development. Consistent with the protective role of Nur77 in this setting, Hu and colleagues demonstrated that Nur77 overexpression limits foam-cell formation, inflammation, and atherosclerotic plaque progression in the ApoE-null background [264]. 

The importance of cardiac fibrosis as a hallmark of detrimental remodeling after MI recently generated interest in the pro-fibrotic role of Nur77 [265]. These authors found that Nur77-null mice subjected to MI or chronic isoproterenol infusion have severe myocardial wall thinning and rupture, accompanied by reduced collagen deposition. This study suggested that cardiac-specific Nur77 regulates EMT transition by cardiac fibroblasts through TGFβ-dependent mechanisms [265].

## 10. Clinical Limitations

The pharmacological development of selective RXR agonists (rexinoids) constituted a promising advancement in NR-based therapy in cancer clinics. Bexarotene is an approved treatment against T-cell lymphoma due to its antineoplastic activity [266,267]. However, it provokes undesirable side-effects, such as hypertriglyceridemia [268] and hypothyroidism [269], making it an unacceptable drug for patients with CVD. These adverse events, which are mediated by the pathological activation of permissive heterodimers, evidence the necessity to generate selective cell- and dimer-specific modulators (agonists or antagonists) able to avoid RXR transcriptional promiscuity. For instance, the PPARγ/RXR selective modulator LG101506 improved insulin sensitivity in diabetic rats, without any alterations in thyroid axis or blood triglyceride content [270]. Recently, Di Zhang and colleagues have reported that pyrimidinyl derivatives of rexinoids (bexarotene and LG100268) ameliorated lipidemic side-effects [271], highlighting the feasibility of enhancing the potency and safety of classical rexinoids. Alternative modifications of rexinoids using a nicotinic acid derivative (NEt-TMN analogs) have efficiently resolved yield bottlenecks that were traditionally encountered [272]. Given the fact that the RXR homodimer can modulate gene expression itself, the design of homodimer-specific synthetic ligands, such as LG100754 [273], may establish a valuable approach to bypass unspecific effects. 

Retinoids can also act as potent teratogens, which further limits the clinical application of pan-RXR agonists. Aiming to counteract this effect, many efforts have been made in terms of drug engineering and recently several RXR partial agonists have been successfully modified to reduce their teratogenicity [274].

## 11. Conclusions

Knowledge of the NR family has advanced significantly over recent decades, revealing their importance as transcriptional mediators of CV homeostasis and disease. In particular, RXR partnerships constitute an attractive regulatory hub that orchestrates cardiac physiology. The immense advances in genome-wide techniques raise the possibility of a more robust transcriptomic and epigenetic analysis needed to fully define not only the impact of each receptor individually but also their collective function. Substantial advances have been made in describing NR function, but the cistromes of each NR in the heart are still undefined. Studies to resolve these questions would be of invaluable help in devising pharmacological strategies for CVD. Genome-wide associated studies also have the potential to uncover NR-related SNPs in cardiac conditions that would represent major progress toward personalized therapy. There is also a need to develop cell-specific synthetic RXR/NR modulators that avoid the major side-effects associated with current approaches. Intense research into RXR/NR biology will likely lead in the near future to the development of safer and more effective therapeutic approaches against CVD.

## Figures and Tables

**Table 1:** RXR partnership biology in CV homeostasis and disease. RXRs, Retinoid X Receptors. RARs, Retinoic Acid Receptors. PPARs, Peroxisome proliferator-activated receptors. LXRs, Liver X Receptors. TRs, Thyroid Hormone Receptors. VDRs, Vitamin D Receptors. DHA, docosahexaenoic acid. vLDL, very low-density lipoprotein. HF, heart failure. DiCM, diabetic cardiomyopathy. MI, myocardial infarction. RAAS, renin-angiotensin system. ECM, extracellular matrix. Tregs, regulatory T-cells. LC-FA, long-chain fatty acid.

Nuclear Receptor	Endogenous Ligands	Synthetic Ligands	Role in Cardiac Homeostasis		Role in Cardiac Disease
RXRs	9-*cis* retinoic acid, DHA [20], LC-FA C24:5 [21]	LGD1069 (Bexarotene), LG100268	Heart morphogenesis along with RARs [22]		Reduction of cardiomyocyte stress and apoptosis [23,24,25]
					Modulation of fatty acid metabolism during HF [26]
					Protection against DiCM by promoting glucose tolerance and reducing myocardial fibrosis [27,28]
					Anti-hypertrophic and anti-atherogenic activity [29,30]
RARs	all-*trans* retinoic acid	AM580, AGN193836, AGN195183	Heart tube looping and epicardial cell function during development [22,31,32]		Reduction of cardiomyocyte stress and apoptosis [33]
					Beneficial for MI by inhibiting cardiofibroblast proliferation [34]
					Protection against cardiac remodelling in HF [35]
					Prevention of calcification in smooth muscle cells [36]
PPARs	Unsaturated fatty acids, eicosanoids, fatty acid ethanolamides and vLDL	Fibrates, Thiazolidinediones	PPARα	Lipid metabolism homeostasis, cardiomyocyte maturation [37]	Regulation of lipid metabolism upon injury which influences oxidative stress in a disease-dependent manner [38,39,40,41]
					Anti-atherogenic activity in myeloid cells [42]
			PPARδ/ PPARγ	Fatty acid homeostasis [43,44]/regulation of inflammatory processes [45]	Anti-inflammatory activity in macrophages and providing protection for atherosclerosis [46,47,48] and MI [49]
					Anti-inflammatory activity in cardiomyocytes preventing from hypertrophy [50]
					Amelioration of DiCM by diverse signalling pathways [51,52,53]
LXRs	Oxysterols	GW3965, T0901317	Cholesterol and lipid metabolism [54]		Atherosclerosis protection both in macrophages [55] and endothelial cells [56]
					Reduction of oxidative stress and apoptosis in cardiomyocytes [57,58,59] and metabolic regulation in DiCM [60,61,62]
TRs	Triiodothyronine, thyroxine	GC-1, KB141, Dimit [63]	Modulation of ion homeostasis to support cardiac contraction [64,65,66,67]		Protective role against post-ischemic injury [68,69]
VDRs	1,25 vitamin D3	Calcidiol, calcitriol, doxercalciferol [70]	RAAS modulation [71,72] and physiological hypertrophy regulation [73,74,75]		Anti-fibrotic functions by modulation of ECM mediators [76], which correlates with MI outcome [77]
					Anti-atherogenic activity in macrophages [78,79] and other immune cells such as Tregs [80]
Nur77	Unknown	6-mercaptopurine (6-MP) [81]	Modulation of physiological hypertrophy and calcium signalling [18]		Anti-hypertrophic [82,83,84] and anti-apoptotic effects [85,86]
					Protection against vascular injury [87,88,89]
					Anti-atherogenic activity modulating inflammation in macrophages [90]
